# Neutrophil Extracellular Trap (NET)-Mediated Killing of *Pseudomonas aeruginosa*: Evidence of Acquired Resistance within the CF Airway, Independent of CFTR

**DOI:** 10.1371/journal.pone.0023637

**Published:** 2011-09-01

**Authors:** Robert L. Young, Kenneth C. Malcolm, Jennifer E. Kret, Silvia M. Caceres, Katie R. Poch, David P. Nichols, Jennifer L. Taylor-Cousar, Milene T. Saavedra, Scott H. Randell, Michael L. Vasil, Jane L. Burns, Samuel M. Moskowitz, Jerry A. Nick

**Affiliations:** 1 Department of Medicine, National Jewish Health, Denver, Colorado, United States of America; 2 Department of Pediatrics, National Jewish Health, Denver, Colorado, United States of America; 3 Division of Pulmonary Sciences and Critical Care Medicine, Department of Medicine, University of Colorado Denver School of Medicine, Anschutz Medical Campus, Aurora, Colorado, United States of America; 4 Department of Pediatrics, University of Colorado Denver School of Medicine, Anschutz Medical Campus, Aurora, Colorado, United States of America; 5 Cystic Fibrosis/Pulmonary Research and Treatment Center, University of North Carolina, Chapel Hill, North Carolina, United States of America; 6 Department of Microbiology, University of Colorado Denver School of Medicine, Anschutz Medical Campus, Aurora, Colorado, United States of America; 7 Division of Pediatric Infectious Disease, Department of Pediatrics, Seattle Children's Hospital, University of Washington School of Medicine, Seattle, Washington, United States of America; 8 Department of Pediatrics, Massachusetts General Hospital and Harvard Medical School, Boston, Massachusetts, United States of America; Louisiana State University, United States of America

## Abstract

The inability of neutrophils to eradicate *Pseudomonas aeruginosa* within the cystic fibrosis (CF) airway eventually results in chronic infection by the bacteria in nearly 80 percent of patients. Phagocytic killing of *P. aeruginosa* by CF neutrophils is impaired due to decreased cystic fibrosis transmembrane conductance regulator (CFTR) function and virulence factors acquired by the bacteria. Recently, neutrophil extracellular traps (NETs), extracellular structures composed of neutrophil chromatin complexed with granule contents, were identified as an alternative mechanism of pathogen killing. The hypothesis that NET-mediated killing of *P. aeruginosa* is impaired in the context of the CF airway was tested. *P. aeruginosa* induced NET formation by neutrophils from healthy donors in a bacterial density dependent fashion. When maintained in suspension through continuous rotation, *P. aeruginosa* became physically associated with NETs. Under these conditions, NETs were the predominant mechanism of killing, across a wide range of bacterial densities. Peripheral blood neutrophils isolated from CF patients demonstrated no impairment in NET formation or function against *P. aeruginosa*. However, isogenic clinical isolates of *P. aeruginosa* obtained from CF patients early and later in the course of infection demonstrated an acquired capacity to withstand NET-mediated killing in 8 of 9 isolates tested. This resistance correlated with development of the mucoid phenotype, but was not a direct result of the excess alginate production that is characteristic of mucoidy. Together, these results demonstrate that neutrophils can kill *P. aeruginosa* via NETs, and *in vitro* this response is most effective under non-stationary conditions with a low ratio of bacteria to neutrophils. NET-mediated killing is independent of CFTR function or bacterial opsonization. Failure of this response in the context of the CF airway may occur, in part, due to an acquired resistance against NET-mediated killing by CF strains of *P. aeruginosa*.

## Introduction

Cystic fibrosis (CF) is the most common lethal genetic disease to affect the non-Hispanic white population in the United States [1], [2]. Despite advances in treatment [3], pulmonary complications remain the leading cause of death [4]. While increased inflammation is present in CF infants [5], the eventual development of chronic airway infection with *Pseudomonas aeruginosa* is associated with an accelerated decline in lung function and increased morbidity and mortality [6], [7], [8], [9], [10], [11], [12], [13].

Neutrophils provide the first line of defense against airway infection by killing and digesting phagocytosed bacteria and fungi. The CF airway contains abundant neutrophils [14], which may contribute to clearance of initial exposures to *P. aeruginosa* [13]. Over time, the neutrophil fails to eradicate *P. aeruginosa,* and the dysregulated release of intracellular components plays a significant role in accelerating the development of bronchiectasis. Dysfunction of the CF neutrophil occurs both as a result of the intense inflammatory and proteolytic milieu within the CF airway, and as a direct result of decreased cystic fibrosis transmembrane conductance regulator (CFTR) expression within the cell [15], [16], [17], [18], [19], [20], [21], [22]. In particular, lack of CFTR function has been linked to decreased phagocytic capacity via reduced intraphagolysosomal HOCl production, resulting in defective killing of *P. aeruginosa* [16], [23], [24]. However, other key elements of the antimicrobial response, including reactive oxygen species generation via NADPH oxidase components appear normal, independent of CFTR function in the neutrophil [25].

Failure of CF neutrophils to eradicate *P. aeruginosa* also results from adaptation of the pathogen to resist host defenses within the unique environment of the CF lung. *P. aeruginosa* displays hypermutability in the CF airway [26], [27], facilitating the expression of virulence determinants postulated to contribute to chronic infection [26], [28], [29], [30], [31], [32], [33], [34], [35]. Among these, the development of mucoidy is one of the most commonly observed phenotypes among CF airway isolates of *P. aeruginosa*, which impairs phagocytic killing by neutrophils [36] and is linked to chronic infection and accelerated airway injury [37], [38], [39], [40].

The identification of neutrophil extracellular traps (NETs) as an alternative mechanism of bacterial killing prompts careful consideration of their role in the CF airway. NETs are extracellular structures comprised of neutrophil chromatin complexed with granule proteins [41]. NETs bind and kill pathogens by juxtaposing microbes with neutrophil granule proteins and histones [41], [42]. The formation of NETs involves a distinct mechanism of neutrophil death [43], though pathways not leading to death exist [44], [45]. Little is known about the signaling mechanisms that trigger NET formation. While in early experimental designs intact NADPH oxidase function, myeloperoxidase, and neutrophil elastase appeared essential [43], [46], [47], early NET formation against *S. aureus* can occur independent of NADPH oxidase [45]. NETs appear to play a protective role in many infections, including appendicitis, shigellosis, Group A *Streptococcus* (GAS) soft tissue infections and pharyngitis, pneumococcal pneumonia, and sepsis [41], [48], [49]. The relevance of NETs to human disease is supported by the finding that GAS strains that express nucleases capable of destroying NETs display enhanced virulence [48], [49].

Many of the current assumptions concerning the role and regulation of NETs arise from the experimental design of the pioneering reports in this rapidly evolving field. Most studies have utilized an activating or priming agent such as phorbol 12-myristate 13-acetate (PMA), chemokines, or cytokines to induce NETs [41], [48], [50], [51], [52], [53], though some pathogens have been shown to directly stimulate NET formation [45], [49], [54], [55], [56]. Initial reports suggested that NETs were fragile [41], so most investigations employed assays with neutrophils motionless on plates [41], [48], [54], [56]. Recently, NETs were shown to remain intact and bind bacteria under shear stress consistent with physiologic flow in the microvasculature [57]; however, the capacity of NETs to kill under the non-stationary conditions present in the circulation or the lung has not been demonstrated *ex vivo*. It is increasingly evident that significant variability exists between types of bacteria with regards to their capacity to evoke NET formation [45] and their susceptibility to NET-mediated killing [48], [49].

We hypothesized that NET-mediated killing of *P. aeruginosa* is impaired in the context of CF airway infection. We tested NET formation and NET-mediated killing across a broad range of multiplicity of infection (MOI), under conditions where the neutrophils and *P. aeruginosa* are maintained stationary on a surface, or are in suspension owing to constant motion. Under conditions resulting in optimal NET-mediated killing, we tested the role of CFTR in NET formation, and the effect of *P. aeruginosa* adaptation over time in the CF airway on susceptibility to NET-mediated killing. Herein we demonstrate that *P. aeruginosa* induces NET formation, and is effectively bound and killed by NETs. Unlike phagocytic killing, NET-mediated killing is not diminished in the absence of functional CFTR. However, as *P. aeruginosa* adapts to the CF airway, it appears to acquire resistance to NET-mediated killing that is independent of alginate overproduction (i.e., mucoidy).

## Materials and Methods

### Ethics statement

These studies were approved by the National Jewish Health Institutional Review Board and written informed consent approved by the National Jewish Health Institutional Review Board was obtained from all neutrophil donors.

### Neutrophil isolation

Human peripheral blood neutrophils were isolated from healthy volunteer donors or CF patients (confirmed by sweat chloride and genetic testing) utilizing the plasma/Percoll method [58]. Whole blood (40 ml) was collected from donors into 50 ml tubes containing 4.4 ml of 3.8% citrate (Fisher Scientific). Samples were maintained at room temperature throughout the isolation procedure to prevent nonspecific neutrophil activation. Tubes were centrifuged at 300 *g* for 20 minutes, with the centrifuge allowed to slow without braking. The platelet-rich plasma layer was aspirated into a fresh tube and centrifuged for 15 minutes at 2500 *g*; the supernatant was removed to obtain platelet-poor plasma (PPP). To the remaining original tube contents (consisting of erythrocytes and leukocytes), 5 ml of 6% dextran (Pharmacia) was added, followed by sufficient 0.9% saline to produce a final volume of 50 ml. Tube contents were mixed by gentle inversion five times, and allowed to stand for 30 minutes at room temperature to allow erythrocyte sedimentation. The leukocyte-rich upper layer was carefully aspirated into a fresh 50 ml tube and centrifuged at 275 *g* for 6 minutes. The resulting pellet was resuspended in 2 ml of PPP, and transferred to a 15 ml polystyrene tube. The leukocyte suspension was then underlayered with 2 ml of 42% Percoll (Pharmacia) freshly prepared in PPP, and both layers then underlayered with 2 ml of freshly prepared 51% Percoll in PPP. The resulting gradients were centrifuged for 10 minutes at 275 *g*. Mononuclear cells and remaining platelets, located at the interface between the upper layer and the 42% Percoll layer, were aspirated into a new tube using a polyethylene transfer pipette. Neutrophils were collected from the interface between the 42% and 51% Percoll layers. The collected neutrophils were then washed once in PPP with centrifugation for 6 minutes at 275 *g*, washed again in Krebs-Ringer phosphate buffer (KRPD) (1.2 mM MgSO_4_, 120 mM NaCl, 5 mM KCl, 1 mM CaCl_2_, 10 mM glucose, 3 mM NaH_2_PO_4_, 12 mM Na_2_HPO_4_), and resuspended at a concentration of 10^7^ neutrophils/ml in KRPD until use. This preparation method yields a >97% pure population of neutrophils.

### Media for all experiments

All experiments were performed in RPMI medium supplemented with 10 mM HEPES (pH 7.4) and 2% heat-inactivated platelet poor pooled human plasma (HIPPP). HIPPP was prepared by pooling the PPP obtained during neutrophil isolation (above) from 5–10 donors. Approximately 300 ml of pooled PPP was placed in a 500 ml polystyrene bottle and incubated in a 56°C water bath for 30 minutes with swirling every 10 minutes, followed by centrifugation at 2500*g* to clear precipitated proteins. Aliquots (1 ml) of HIPPP were stored for up to 6 months at −20°C before use. The experimental media was confirmed to contain no detectable DNase activity, which has been detected under experimental conditions that utilize higher concentrations of fetal calf serum [59].

### NET formation assay

During initial studies of NET formation, we observed extensive variability in NET production by unstimulated neutrophils. This appears related to neutrophil-surface interactions or homotypic interactions between neutrophils, as maintaining neutrophils in suspension prevents significant NET formation by unstimulated cells. Accordingly, we utilized a modification of the method of Fuchs *et al*. for NET quantitation [43]. Purified human neutrophils (10^6^ in 200 µl of the media above) were treated with 25 nM PMA (Sigma), 10 µM DPI (Sigma) or bacteria at the indicated MOI at 37°C with rotation at 8 rpm. At each time interval tested, limited nuclease digestion was performed with micrococcal nuclease (0.5 units/ml for 10 minutes at 37°C)(Sigma). Nuclease activity was then stopped with 5 mM EDTA (Sigma), and cellular debris removed by centrifugation. DNA content was measured with the Quant-iT™ Picogreen assay (Invitrogen).

### Bacteria

*P. aeruginosa* PAO1 was obtained from the Pseudomonas Genetic Stock Center (East Carolina University). Deidentified, isogenic-paired early and late clinical isolates of *P. aeruginosa* were obtained from the laboratories of Michael L. Vasil, Jane L. Burns and Samuel M. Moskowitz [13], [60], [61], [62]. A *mucA* mutant of PAO1 generated by targeted disruption with a gentamicin resistance cassette, and a transposon-generated *mucA* mutant of PAO1 (PW2387) was obtained from the University of Washington *P. aeruginosa* mutant library [63]. All bacteria were grown on LB agar or in LB broth. Stationary phase bacteria were utilized for all experiments. For experiments using opsonized bacteria, PAO1 was opsonized in 0.9% saline containing 20% pooled human serum for 30 minutes in a 37°C incubator with rotation at 8 rpm. Opsonized bacteria were washed and resuspended twice in PBS, and then resuspended in the experimental media (described above). The *Staphylococcus aureus* strain used was a deidentified clinical strain isolated from a CF patient by the Microbiology Laboratory at National Jewish Health.

### Fluorescence microscopy

Stationary phase *P. aeruginosa* PAO1 were washed with saline and resuspended at 1×10^9^ CFU/ml and stained for 10 minutes at 37°C with 10 µg/ml Polymyxin B-BODIPY (Invitrogen) in saline. After washing, labeled bacteria were incubated with neutrophils for 120 minutes at 37°C with rotation at 9 rpm in RPMI medium supplemented with 10 mM HEPES (pH 7.4) and 2% HIPPP. Extracellular DNA was stained with the Sytox Orange (Invitrogen) at a concentration of 0.1 µM. The cell suspension was imaged in micro chamber slides (Ibidi) or, after fixation with 4% paraformaldehyde (Sigma), on glass slides. Microscopy was performed with a Zeiss Axiovert 200M with Slidebook 5 software (Intelligent Imaging Innovations).

### Bacterial killing assays

Using bacterial killing assays in plates [41], [48], [54], we found that, without inhibition of phagocytosis with cytochalasin D, a minor fraction of *P. aeruginosa* killing was NET-mediated (data not shown). We then investigated conditions that maximized NET killing without inhibition of phagocytosis. We found that the majority of killing was attributable to NETs if the neutrophils and bacteria remained in suspension by continuous rotation. For plated killing assays, 10^6^ neutrophils in 200 µl of media (above) were seeded into 96 well plates. Samples were untreated or treated with 25 nM PMA (Sigma) for 105 minutes at 37°C, with or without DNase I at 100 units/ml (Worthington) to degrade NETs. Phagocytic killing was inhibited in some samples by the addition of 100 µg/ml cytochalasin D (Sigma) for 15 minutes before the addition of bacteria. Bacteria at the indicated MOI were added and the plates centrifuged at 800 *g* for 10 minutes. After one hour at 37°C, neutrophils and clumped NETs were disrupted by the addition of 0.01% Triton X-100 and three passes through a 25 gauge needle. Following serial dilution, bacteria were plated on LB plates for enumeration of colonies. Suspension killing assays were performed similarly, except assays were performed in microfuge tubes with rotation at 8 rpm, and no centrifugation was performed. Because neutrophils incapable of killing appeared to enhance bacterial recovery, “zero killing” was defined by control samples consisting of neutrophils treated with DNase I (100 units/ml) to inhibit NET-mediated killing, with and without cytochalasin D (100 µg/ml) as an inhibitor of phagocytic killing. Killing by NETs was determined by subtracting the extent of killing in the presence of DNase I (*i.e.* phagocytic killing) from total killing.

### Statistical analyses

Data were analyzed with GraphPad Prism 4.0 software (GraphPad Software, Inc.) with use of Student's t-test or ANOVA with Bonferroni's post-test as appropriate.

## Results

### *P. aeruginosa* induces NET formation by neutrophils in suspension

**Figure 1:**
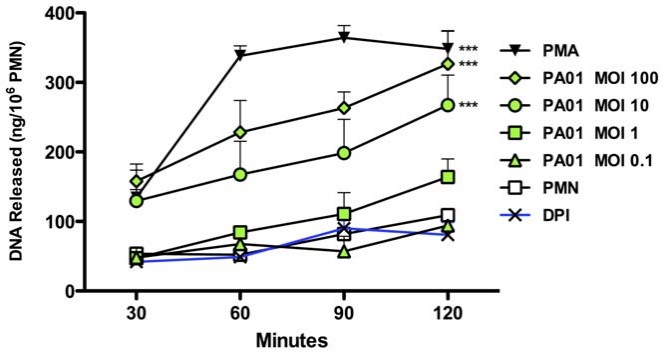
*P. aeruginosa* stimulates NET formation in nonadherent neutrophils. Human neutrophils suspended in media were untreated or treated with 25 nM PMA, 10 µM DPI, or *P. aeruginosa* at MOI of 0.1, 1, 10, or 100. NET formation was measured at 30-minute intervals by DNA released (ng per 10^6^ PMN). Neutrophils were isolated from healthy donors (n = 3) with samples performed in duplicate; error bars represent SEM. ***  =  p<0.001 by two-way ANOVA with Bonferroni's post-test compared to untreated control.

While *P. aeruginosa* has been noted to induce NET formation [45], [64], systematic studies with this bacterium are not available [49], [51], [56], [65], [66]. To date, *in vitro* quantification of NET formation has nearly always been performed with neutrophils seeded onto surfaces; however, we have observed a high background of non-specific NET formation under those conditions (data not shown). With the cells in suspension, we evaluated the capacity of unprimed neutrophils to spontaneously form NETs, and the response of neutrophils to PMA (a potent inducer of NETs [41]), diphenyleneiodonium (DPI) (an inhibitor of NETs [43]), and *P. aeruginosa* strain PAO1 across a four-log_10_ range of MOI [67]. Under these conditions, little release of NETs was detected over 2 hours by untreated neutrophils (Figure 1). *P. aeruginosa* strain PAO1 effectively stimulated NET formation in a bacterial density-dependent fashion (Figure 1).

### NET-mediated killing of *P. aeruginosa* by neutrophils in suspension

**Figure 2:**
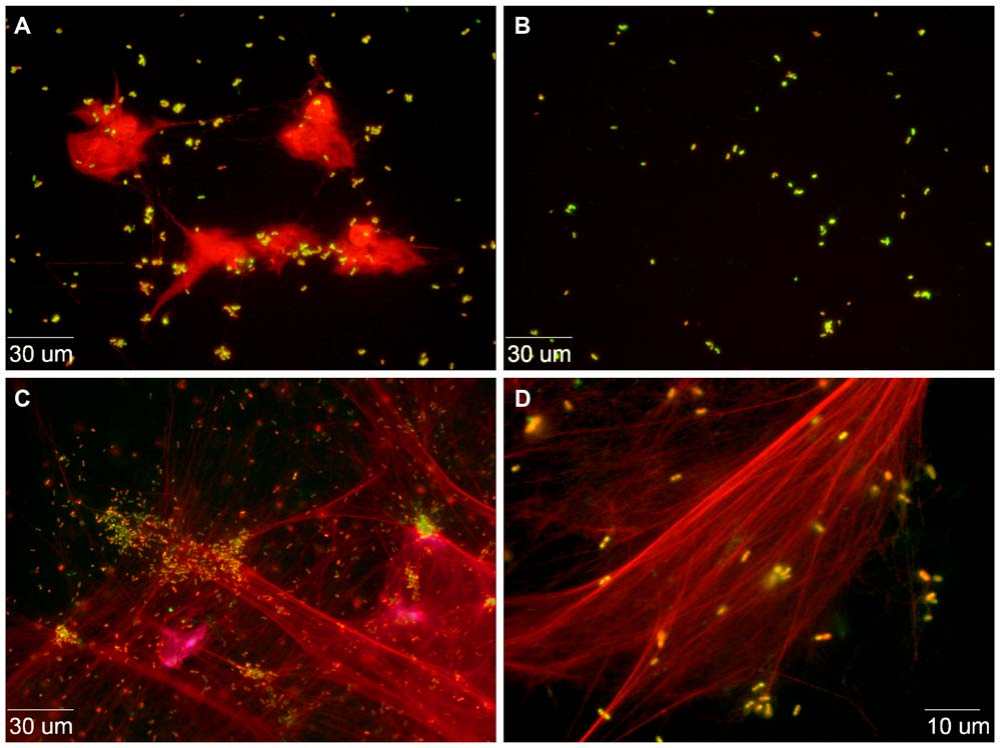
NET formation and binding of *P. aeruginosa* by nonadherent neutrophils. **Panels A and B**: Bacterial cells of strain PAO1 labeled with polymyxin B-BODIPY were incubated with isolated human neutrophils (MOI of 10) for 2 hours with (Panel B) or without (Panel A) DNase. NETs were then stained with the cell-impermeant DNA binding dye, Sytox Orange. In the presence of DNase, NETs are seen to be completely degraded. **Panels C and D** present the results of a similar experiment performed at a higher ratio of bacteria to neutrophils (MOI of 100), to highlight the physical association of bacteria with NETs.

NET-mediated killing occurs through binding of pathogens to strands of DNA to facilitate direct contact of the microbe with antimicrobial neutrophil granule products [41]. Fluorescence microscopy was utilized to evaluate physical entrapment of *P. aeruginosa* within NETs. Neutrophils suspended in media were exposed to fluorescently labeled *P. aeruginosa* strain PAO1 at an MOI of 10 and stained with a cell-impermeant DNA stain, revealing abundant NETs formation (Figure 2A). Confirmation that the observed structures represent NETs was obtained by their absence when the identical experiment was conducted in the presence of DNase (Figure 2B) [41]. Similar experiments performed at an MOI of 100 to optimize visualization of the NETs and bacteria demonstrate physical entrapment of PAO1 within NETs (Figure 2C and 2D).

**Figure 3:**
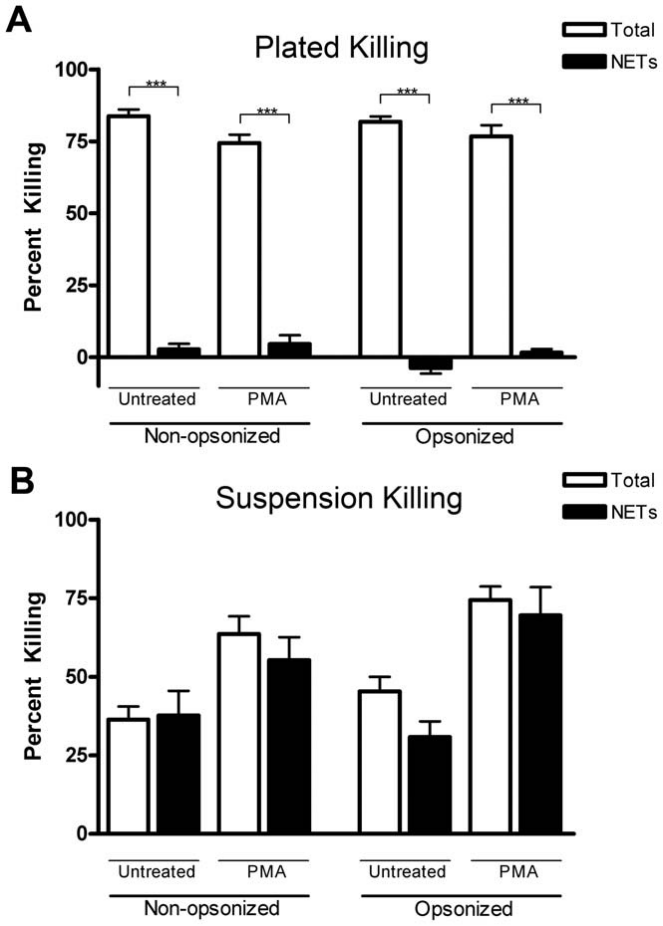
NET-mediated killing of *P. aeruginosa* is most effective for nonadherent neutrophils. **Panel A**: Non-opsonized or opsonized bacterial cells of strain PA01 were juxtaposed to neutrophils on the surface of a plate by centrifugation (MOI of 0.01). Neutrophils were untreated or exposed to 25 nM PMA for 2 hours to maximize NET formation prior to the addition of bacteria. ***  =  p<0.001 and NS  =  p>0.05 by ANOVA with Bonferroni's post-test. P**anel B**: This killing assay was performed with continuous rotation of the neutrophils. Under these conditions, virtually all killing is attributable to NETs. Neutrophils were isolated from healthy donors (n = 5) with samples performed in duplicate; error bars represent SEM. All comparisons were non-significant, with p>0.05 by ANOVA with Bonferroni's post-test.

Close physical association between pathogens and neutrophils on a motionless surface is the standard experimental design for assays of NET-mediated killing [41], [48], [54]. However, surface adherence of neutrophils can activate integrin-mediated signaling pathways, and modify a broad range of responses [68]. We investigated the role of surface contact on killing of *P. aeruginosa*. Using a typical assay for NET-mediated killing, in which bacteria and neutrophils are layered together on a surface by centrifugation, phagocytic killing (*i.e.* DNase resistant) is the primary mechanism by which neutrophils kill PAO1, evidenced by the fact that only a small fraction of total killing is attributable to NETs (Figure 3A). In contrast, when neutrophils and *P. aeruginosa* are incubated together in a suspended state, nearly all killing is NET-mediated (Figure 3B). Non-specific opsonization of bacteria did not enhance non-NET-mediated killing by stationary neutrophils (Figure 3A), and likewise was not required for NET-mediated killing in suspension (Figure 3B). As PAO1 stimulates NET formation (Figure 1), NET-mediated killing of the bacteria can occur in the absence of additional activation of the neutrophil (Figure 3B). Greater NET-mediated killing can be achieved by inducing maximal NET release in response to PMA, followed by introduction of *P. aeruginosa* (Figure 3B). Of note, this strong dependence on NET-mediated killing in the suspended state occurred optimally under conditions in which a low concentration of HIPPP (2%) was present in the media. Previously, it has been reported that higher concentrations of serum inhibit NET formation in a concentration-dependent fashion [43], and we confirmed in this system that virtually no killing of *P. aeruginosa* in suspension is NET-mediated when higher concentrations of serum are present (data not shown).

### NET-mediated killing of *P. aeruginosa* occurs over a broad range of MOI

**Figure 4:**
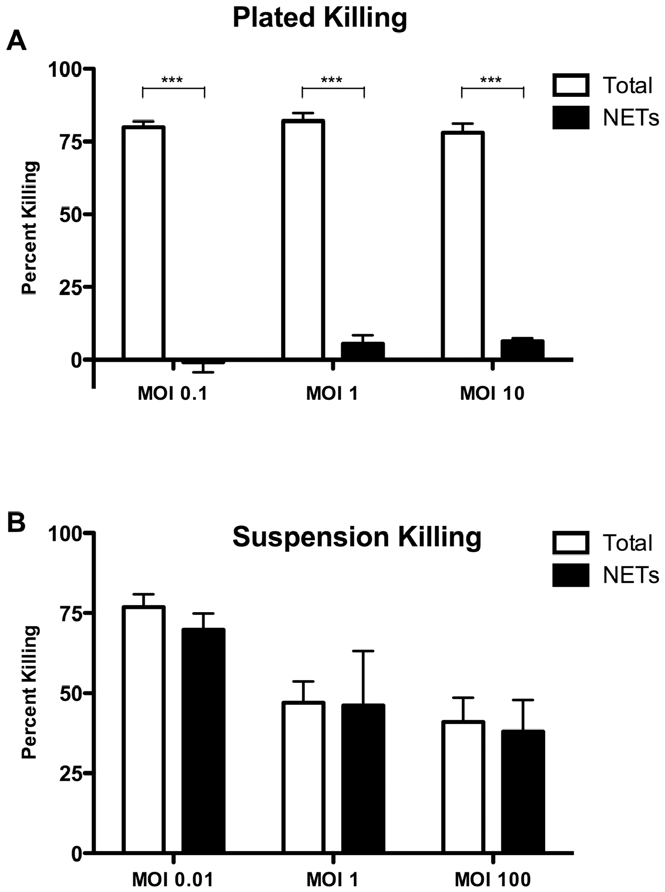
NETs kill *P. aeruginosa* in suspension across a wide range of MOI. **Panel A**: The effect of MOI on NET-mediated killing was tested with neutrophils and PAO1 stationary on a plate, as depicted in Figure 3A. Over a 3-log range of MOI, NET-mediated killing represented a very small fraction of total killing. ***  =  p<0.001 and NS  =  p>0.05 by ANOVA with Bonferroni’s post-test. **Panel B**: With PAO1 and neutrophils in suspension, NET-mediated killing accounted for nearly all killing over a 5-log range of MOI. The lowest MOI tested trended towards greater efficiency of NET-mediated killing. Neutrophils were isolated from healthy donors (n = 5) with samples performed in duplicate. Differences in total killing and NETs killing at each MOI were not significant.

Some studies suggest that phagocytic killing of *P. aeruginosa* and other bacteria by neutrophils varies in efficiency with changes in MOI [69], [70], though this has been debated [32]. Thus, we tested the effect of MOI on *P. aeruginosa* PAO1 killing under conditions optimized for either phagocytic or NET-mediated killing. Neutrophils stationary on a surface (see Methods) were activated with PMA to induce maximal NET formation, and were then incubated with PAO1 at MOI from 0.1 to 10. NET-mediated killing was significantly less than phagocytic killing for all bacteria to neutrophil ratios (Figure 4A). With cells in suspension, NET-mediated killing was most efficient at the lowest MOI tested (0.01), although greater absolute numbers of bacteria were killed at higher MOIs (Figure 4B). As seen in Figure 3B, NET-mediated killing was the predominant mechanism by which neutrophils killed PAO1 under conditions of constant motion.

### Neutrophils isolated from CF patients display normal NET-mediated killing of *P. aeruginosa*

**Figure 5:**
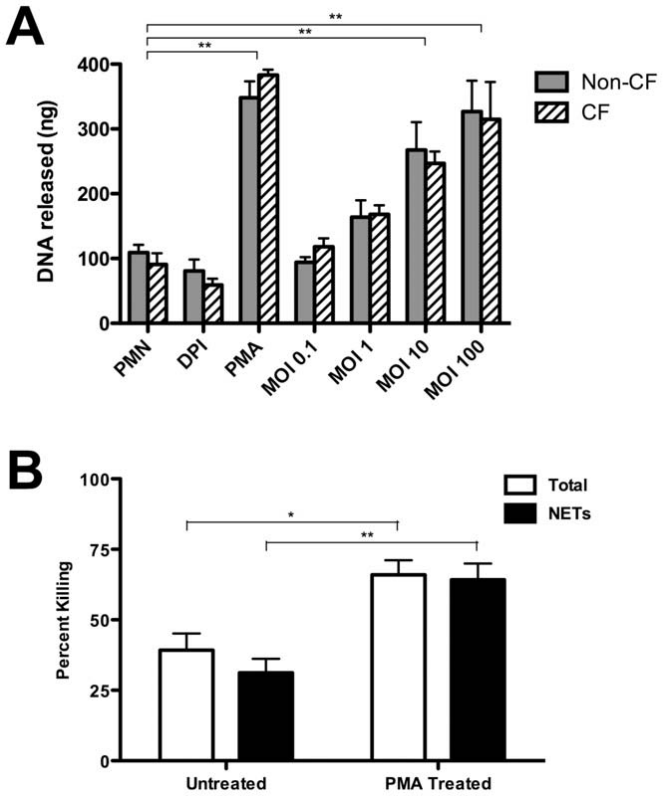
Neutrophils isolated from CF patients form NETs that effectively kill *P. aeruginosa*. **Panel A**: Neutrophils from healthy volunteers (filled boxes) or CF patients (hatched boxes) were unstimulated (PMN), treated with an inhibitor of NET formation (DPI), treated with a stimulant of maximal NET formation (PMA), or exposed to PAO1 at the indicated MOI. Released NET-associated DNA was quantitated after a 120-minute exposure. There were no significant differences in NET formation between neutrophils isolated from healthy volunteers and CF patients. **Panel B**: CF neutrophil NET-mediated killing of bacterial cells of strain PAO1 was performed in suspension as in Figure 3B. Effective killing of PAO1 was observed in the absence of additional stimulation, and was significantly enhanced by pre-treatment with PMA to stimulate maximal NET formation. For both panels, neutrophils were isolated from CF patients (n = 5) with samples performed in duplicate; error bars represent SEM. *  =  p<0.05 and **  =  p<0.01 by ANOVA with Bonferroni’s post-test.

Recently, defects in phagocytic killing of *P. aeruginosa* have been linked to decreased HOCl production within the neutrophil phagolysosome, as a direct result of absent or reduced CFTR function [16], [23], [24]. NET production and bactericidal activity of neutrophils isolated from CF patients were evaluated separately in response to *P. aeruginosa*. CF neutrophils had nearly identical baseline NET-formation, response to PMA, and concentration-dependent response to *P. aeruginosa* as neutrophils isolated from healthy donors (Figure 5A). In addition, the NETs formed by CF neutrophils effectively killed PAO1 (Figure 5B).

### Clinical strains of *P. aeruginosa* develop resistance to NET-mediated killing within the CF airway

To better evaluate the significance of NET-mediated killing of *P. aeruginosa* in the CF airway, we examined whether clinical isolates of *P. aeruginosa* were effectively killed by this mechanism. Nine well-characterized paired isolates of *P. aeruginosa* from CF patients were utilized (Tables 1 and 2), with an “early” isolate recovered at or shortly after initial infection, and a second “late” isogenic isolate recovered at a later date [13], [60], [61], [62]. The mean interval between isolation of the strains was 10.6 years (range 0.25–18 years). In 8 of 9 pairs, the early isolate had a non-mucoid phenotype, while all of the late isolates had converted to a mucoid phenotype. In addition to mucoidy, changes in colony size, shape and color were evident, along with acquisition of changes in resistance to standard anti-pseudomonal antibiotics (Tables 1 and 2). Growth rates also demonstrated considerable divergence between early and late isolates, as 7 of 9 late strains grew more slowly under the conditions tested, with longer time in lag phase, and greater time until mid-log phase (Table 1).

**Table 1:** Growth characteristics of early and late CF clinical isolates of Pseudomonas aeruginosa.

Strain			Morphology				Growth		
ID number	Isolate^1^	Isolation Date	Mucoid	Colony Size	Colony Shape	Color	Lagtime^2^ (min)	V_50_ 3 (min)	DNA release^4^ (ng/10^5^ bacteria)
AMT0009	**E**	Mar-97	N	large	irregular		520	674	7.2±0.8
	**L**	Apr-08	Y	small	round	translucent	848	1182	10.8±1.0^NS^
AMT0105	**E**	Sep-93	N	very small	round	yellowish tan	569	696	26.3±1.9
	**L**	Jul-06	Y	small	round	pink & tan	621	875	43.3±1.5^NS^
AMT0145	**E**	Jan-90	N	small	irregular		646	960	14.2±0.8
	**L**	Jan-02	Y	small	round		936	1504	16.1±0.5^NS^
AMT0147	**E**	Feb-95	N	small to medium	round		505	591	12.4+1.1
	**L**	Jan-96	Y	very small	round	pinkish	630	824	11.9±3.4^NS^
AMT0027	**E**	Jun-97	N	small	Irregular	pinkish tan	510	780	7.0±0.8
	**L**	Aug-09	Y	small	round	tan	555	764	13.9±2.4^NS^
AMT0058	**E**	Dec-94	N	small	round	slightly metallic	732	1016	7.1±1.1
	**L**	Oct-09	Y	small	round	pinkish tan	838	1056	13.5±0.6^NS^
AMT0059	**E**	Sep-91	Y	small	round	pinkish tan	720	1003	12.4±0.8
	**L**	Aug-09	Y	small	round	pinkish tan	600	831	17.3+0.6^NS^
AMT0294	**E**	Apr-96	N	small	round	rings	805	1053	15.2±0.6
	**L**	Aug-09	Y	small	round	pinkish tan	615	1150	12.8±0.4^NS^
CO CF1	**E**	Feb-86	N	small	round	yellow	420	570	15.1±0.4
	**L**	May-86	Y	small	round	yellow	750	1012	12.6±1.1^NS^
PA01	**N/A**	N/A	N	small	round	yellow	482	568	14.6±0.8

1 Isolate: E indicates an isolate recovered early after infection, L indicates isolate recovered later in the life of the patient.

2 Lagtime: Time (minutes) from start of culture to start of log phase growth.

3 V_50_: Time (minutes) from start of culture to midpoint of log phase growth.

4 DNA release: Quanitity of DNA released from each isolate under the conditions tested in the NET-induced killing assay.

NS Not Significant compared to isogenic early isolate by Wilcoxon 2-sample test.

**Table 2:** Antibiotic Susceptibility of early and late CF clinical isolates of Pseudomonas aeruginosa.

Strain		Antibiotic Susceptibility^1^								
ID number	Isolate^2^	Amikacin	Aztreonam	Cefepime	Ceftazidime	Ciprofloxacin	Meropenem	Pip/Tazo	Tobramycin	Trimeth/Sulfa
AMT0009	**E**	R (>256)	R (>256)		S (6)	S (0.5)	R (32)	R (256.1)	S (<4)	R (32)
	**L**	R (>256)	R (>256)		S (6)	S (0.5)	R (32)	R (256.1)	S (<4)	R (32)
AMT0105	**E**				S	S				R
	**L**	S (4)	R (>256)		R (>256)	I (1.5)			S	S (2)
AMT0145	**E**	S (1)	R (32)	I (16)	S (4)	I (2)	S (4)	R (256)	S (0.24)	R (128)
	**L**	I (32)	R (32)		I (16)	R (8)	I (8)		S (2)	R (16)
AMT0147	**E**				R (8)	S (2)				R (4)
	**L**				R	R				R
AMT0027	**E**	S (1)	S (0.9)	S (4)	S (1)	S (1)	S (0.49)	S (3.9)	S (0.9)	S (2)
	**L**	S (4)	S (4)	R (32)	S (2)	I (2)	I (8)	R (256)	S (0.9)	R (16)
AMT0058	**E**				R	S				R
	**L**	S (16)	R (32)	R (32)	I (16)	R (4)	I (8)	R (256)	R (128)	R (8)
AMT0059	**E**	S (2)	I (16)	S (8)	S (4)	S (1)	S (1)	S (2)	S (0.9)	R (4)
	**L**	S (4)	I (16)	S (8)	S (4)	I (2)	R (16)	S (8)	S (0.9)	S (2)
AMT0294	**E**	S (4)	R (32)	S (8)	S (8)	S (0.5)	S (2)	I (32)	S (0.5)	R (16)
	**L**	S (8)	S (8)	I (16)	I (16)	S (0.5)	S (1)	S (2)	S (4)	
CO CF1	**E**			S				S	S	S
	**L**			S				S	S	S
PA01	**N/A**	S	S	S	S	S	S	S	S	S

1 Antibiotic susceptibility: R =  resistant, I = intermediate, S =  sensitive, (minimum inhibitory concentration).

2 Isolate: E indicates an isolate recovered early after infection, L indicates isolate recovered later in the life of the patient.

**Figure 6:**
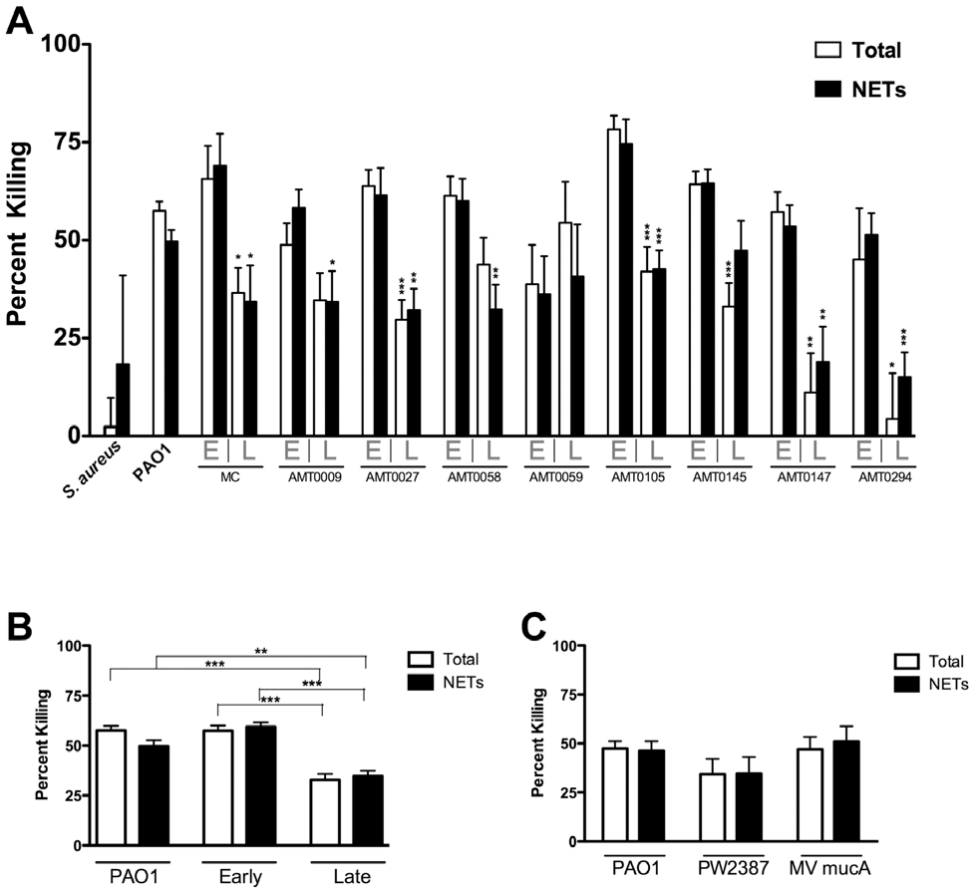
*P. aeruginosa* acquires resistance to NET-mediated killing in the CF airway. **Panel A.** Neutrophils were stimulated with PMA to induce maximal NET formation and were then exposed to isogenic clinical strains of *P. aeruginosa* isolated from CF patients around the time of the first positive culture (“**E**arly”) or a mean of 10.6 years later (“**L**ate”). *  =  p<0.05, **  =  p<0.01, and *** = P<0.001 by Student’s t-test comparing early strain killing versus late strain killing for each pair of isolates. Neutrophils were isolated from healthy donors (n  =  ≥4 for each set) with samples performed in duplicate and error bars represent SEM. Total and NET-mediated killing were determined as in Figure 3. **Panel B**. Aggregate analysis of the data in Panel A demonstrates a significant resistance to NET-mediated killing acquired by the **L**ate isolates when compared to **E**arly isolates, or laboratory-adapted strain PAO1. *  =  p<0.05, **  =  p<0.01, and *** = P<0.001 by ANOVA with Bonferroni’s post-test. **Panel C**. Isolated human neutrophils were stimulated with PMA as for Panel A, and exposed to *P. aeruginosa* PAO1 or two independently derived *mucA* mutants of PAO1, PW2387 (University of Washington *Pseudomonas* transposon mutant library) or MV *mucA* (targeted gentamicin cassette disruption from the laboratory of Michael Vasil) with assessment of total and NET-mediated killing. Neutrophils were isolated from healthy donors (n  =  ≥5) with samples performed in duplicate. Differences between samples in NET-mediated or total killing were not significant by Student’s t-test.

Early CF isolates were killed by NETs with a similar efficiency as PAO1 (Figure 6A). However, in 7 of 9 isolates, a significant reduction in NET-mediated killing was observed for the late isolates. In aggregate, there was a 41.5% reduction in the percentage of *P. aeruginosa* killed via NETs by the late isolates compared to the early isolates (p<0.001)(Figure 6B). A clinical CF isolate of *S. aureus* had a relatively low rate of NET-mediated killing relative to *P. aeruginosa*. To assess whether the reduced susceptibility of the late isolates to killing by NETs was explained solely by excess exopolysaccharide production (which characterizes the mucoid phenotype), we tested two independently derived *mucA* mutants of PAO1, which overexpress alginate, for their susceptibility to NET killing. Neither of the *mucA* mutants displayed a statistically significant difference in sensitivity to killing by NETs in comparison to wild type PAO1 (Figure 6C). Extracellular DNA release, a virulence mechanism for *P. aeruginosa*, was also tested. Overall, the release of DNA from the strains was low, under the conditions of the NET-mediated killing assay. In 6 of 9 pairs, the late isolate released greater DNA, however, this difference did not reach statistical significance in any of the pairs studied (Table 1).

## Discussion

Neutrophil extracellular traps are a recently described mechanism by which neutrophils kill a variety of pathogens. However, significant variability appears to exist in the relative susceptibility to NET-mediated killing by clinically important microbes, and the relative biologic roles of NET versus phagocytic killing have yet to be clearly elucidated in health and disease (i.e., in specific clinical infections or within specific anatomic compartments). Herein, we demonstrate that *P. aeruginosa* induces formation of NETs and is susceptible to NET-mediated killing *ex vivo* under specific conditions. Under conditions typically used to study NETs, where bacteria and neutrophils were layered together on a motionless surface, we found that phagocytic killing of *P. aeruginosa* was the predominant response. In contrast, under conditions in which the bacteria and neutrophils are maintained in suspension with constant motion and mild shear forces, nearly all killing of *P. aeruginosa* was NET-mediated. To our knowledge, these experimental test conditions have not previously been examined. Under these conditions, NET-mediated killing was efficient even with a very low ratio of *P. aeruginosa* to neutrophils and was not dependent on opsonization of the bacteria. This method was optimized to test the parameters of NET-mediated killing of *P. aeruginosa*; optimal NET-mediated killing of other pathogens may require different experimental conditions.

In the context of CF lung disease, we explored the possibility that CF neutrophils might fail to produce effective NETs, prompted by observations that a number of neutrophil responses are abnormal in the setting of non-functional CFTR [16], [17], [18], [19], [20], [21], [22], [23]. Specifically, previous reports have indicated that CFTR deficiency results in reduced phagolysosomal function in neutrophils and macrophages, and impaired phagocytic killing of *P. aeruginosa* [16], [23], [56], [71]. In contrast, our results indicate that CF neutrophils produce functional NETs comparable to those of neutrophils with functional CFTR. Thus, NET-mediated killing is a CFTR-independent arm of the innate immune response that may assume greater importance in CF patients. Despite many reports identifying impaired response by CF neutrophils, these cells clearly have substantial antimicrobial capabilities, as bacterial infections outside the airway are not a feature of the disease. Our findings suggest that NET-mediated killing is fully functional in CF patients, and thus may account for the general absence of invasive infection despite a massive bacterial burden within the CF airway.

Our data suggest a role for NET-mediated clearance of initial *P. aeruginosa* infection in healthy individuals, and possibly in the early stages of CF lung disease. Episodic exposure of humans to *P. aeruginosa* is likely a common occurrence. Even within the CF airway, *P. aeruginosa* is often cleared effectively, and progression to chronic infection may not occur for years [13]. One possibility is that resident macrophages within the lung could be responsible for this initial clearance. However, another possibility is that NET-mediated killing by neutrophils may account for early clearance. Conditions that favor NET-mediated killing are likely present within both CF and normal airways. The lung is in constant motion, and environmental exposures typically involve small inocula of bacteria with limited direct contact with neutrophils, in the presence of low plasma concentrations and without effective opsonization [72]. Consistent with this premise, our results indicate that *P. aeruginosa* strains isolated from CF airways early in the course of infection are effectively killed by NETs.

Strong evidence indicates that neutrophil defenses ultimately fail as CF lung disease progresses and *P. aeruginosa* infection becomes persistent. For CF patients, inhaled DNase therapy improves lung function and reduces infectious exacerbations [4], [73], [74]. Since DNase disrupts killing by NETs [41], these results support the conclusion the NET-mediated killing is not effective within the airway of CF patients who have established infection. Given the evidence suggesting that NETs do not facilitate bacterial clearance later in the course of CF, NET formation may actually be detrimental by promoting hyperviscosity of airway secretions, release of neutrophil proteases, and development of *P. aeruginosa* biofilms [61], [75].

One factor which could contribute to ineffective NET-mediated killing within the CF airway is acquired *P. aeruginosa* resistance to this arm of innate host defense. The hypermutability of *P. aeruginosa* within the CF airway is well-described and it is not surprising that in this intense inflammatory environment, mutants with increased resistance to NETs would emerge [26], [28], [29], [30], [31]. We tested the capacity of CF strains of *P. aeruginosa* to acquire resistance to NET-mediated killing. Using paired isogenic clinical isolates of *P. aeruginosa*, we showed that decreasing susceptibility to NET-mediated killing evolves over time in the CF airway. The development of mucoidy (*i.e.* increased alginate production) is an acquired *P. aeruginosa* virulence factor that is closely associated with acceleration of CF lung disease [37], [38], [39], [40]. Among the nine pairs of isolates tested, conversion to a mucoid phenotype coincided with a decline in susceptibility to NETs, raising the possibility that increased alginate production decreases interactions with NETs, or otherwise interferes with killing by NET-associated granule proteins. However, two independently derived *mucA* mutants of PAO1 failed to display the NET resistance seen for late CF airway isolates, suggesting that increased exopolysaccharide production alone does not explain this phenotype.

Presumably, increased expression of other, as yet unidentified, determinants may act either independently or concurrently with increased alginate production to mediate NET resistance. Several pathogens possess specific mechanisms that disrupt NET-mediated killing, including *Streptococcus pneumoniae* and GAS, which produce nucleases that degrade NETs [48], [49]. In addition, capsule formation, in concert with D-alanylation of lipoteichoic acids, enhances resistance of *S. pneumoniae* to NET-mediated killing [76]. GAS strains expressing the M1 protein resist NET-mediated killing by virtue of their resistance to the human cathelicidin peptide LL-37, an important antimicrobial component of NETs [77]. *P. aeruginosa,* with its extremely large genome, is capable of tremendous versatility and environmental adaptability [78]. It encodes deoxyribonucleases, and the possibility exists that these, or related enzymes, could act on NETs [79], as well as yet undescribed mechanisms of disrupting NET-mediated killing. Although not tested here, extrinsic features of the CF airway could also contribute to ineffective NET function in the setting of chronic *P. aeruginosa* infection. The CF airway is a complex environment characterized by altered airway mucus, high levels of proteases, large amounts of neutrophil-derived DNA and F-actin, and abundant *P. aeruginosa* [80], [81], [82], [83], [84]. One or more of these features may disrupt NET killing by preventing NET formation, disassembling or altering the antibacterial components of NETs, preventing physical interaction between *P. aeruginosa* and NETs, or competing for binding sites on NETs.

We postulate that NETs contribute to early clearance of *P. aeruginosa* from the CF airway, but that, later in the disease, features of the CF airway or an adaptation of the organism render NETs ineffective, and possibly detrimental. If in fact NET-mediated killing is effective in the initial contact between *P. aeruginosa* and neutrophils suspended within the CF airway secretions, this has important implications both for development of new therapies and for early CF airway disease. This notion may also help guide the use of inhaled DNase in other lung conditions for which benefit might be assumed based on results in CF patients. For example, disruption of effective NET killing could explain the increased rates of infection reported with DNase use in non-CF bronchiectasis [85]. Understanding the role of NETs in controlling *P. aeruginosa* at different stages of airway infection is particularly important given ongoing clinical trials evaluating the use of this agent in very young children [86], and in devising strategies to prevent initial infection by enhancing host defenses.
